# Synthetic mimetics of the endogenous gastrointestinal nanomineral: Silent constructs that trap macromolecules for intracellular delivery

**DOI:** 10.1016/j.nano.2016.07.008

**Published:** 2017-02

**Authors:** Laetitia C. Pele, Carolin T. Haas, Rachel E. Hewitt, Jack Robertson, Jeremy Skepper, Andy Brown, Juan Carlos Hernandez-Garrido, Paul A. Midgley, Nuno Faria, Helen Chappell, Jonathan J. Powell

**Affiliations:** a Medical Research Council Human Nutrition Research, Elsie Widdowson Laboratory, Cambridge, UK; b Cambridge advanced Imaging Centre, Physiology development and Neuroscience, Anatomy building, University of Cambridge, Cambridge; c Institute for Materials Research, SCAPE, University of Leeds, Leeds; d Departamento de Ciencia de los Materiales e Ingeniería Metalúrgica y Química Inorgánica, Facultad de Ciencias, Universidad de Cádiz, Campus Universitario Rio San Pedro, Puerto Real, Spain; e Department of Materials Science and Metallurgy, University of Cambridge, Cambridge

**Keywords:** Amorphous magnesium-substituted calcium phosphate, Nanoparticles, Peptidoglycan, PD-L1, ACP, amorphous calcium phosphate, AMCP, amorphous magnesium-substituted calcium phosphate, APC, antigen presenting cell, BSA, bovine serum albumin, Pg, peptidoglycan, LPS, lipopolysaccharides, TEM, transmission electron microscopy, HAADF, high-angle annular dark-field, STEM, scanning transmission electron microscopy, ICP-OES, inductively coupled plasma optical emission spectrometry, TCM, tissue culture medium

## Abstract

Amorphous magnesium-substituted calcium phosphate (AMCP) nanoparticles (75-150 nm) form constitutively in large numbers in the mammalian gut. Collective evidence indicates that they trap and deliver luminal macromolecules to mucosal antigen presenting cells (APCs) and facilitate gut immune homeostasis. Here, we report on a synthetic mimetic of the endogenous AMCP and show that it has marked capacity to trap macromolecules during formation. Macromolecular capture into AMCP involved incorporation as shown by STEM tomography of the synthetic AMCP particle with 5 nm ultra-fine iron (III) oxohydroxide. *In vitro*, organic cargo-loaded synthetic AMCP was taken up by APCs and tracked to lysosomal compartments. The AMCP itself did not regulate *any* gene, or modify any gene regulation by its cargo, based upon whole genome transcriptomic analyses. We conclude that synthetic AMCP can efficiently trap macromolecules and deliver them to APCs in a silent fashion, and may thus represent a new platform for antigen delivery.

## Graphical Abstract

Amorphous magnesium-substituted calcium phosphate (AMCP) nanoparticles, which form naturally in the mammalian gut, may play an important role in immune surveillance by promoting the delivery of luminal macromolecules to mucosal immune cells. To further probe this constitutive phenomenon, we set out to develop a synthetic mimetic generating *in vitro* amorphous calcium phosphate nanoparticles similar to their *in vivo* counterparts. Resulting synthetic particles were of the right size (75-150 nm), phase (amorphous) and composition (magnesium-substituted calcium phosphate) and trapped macromolecules effectively delivering them to antigen presenting cells without adverse effects. The silent delivery of macromolecules by synthetic AMCP informs on their *in vivo* role and could be exploited as a safe platform for antigen delivery.

**Figure:**
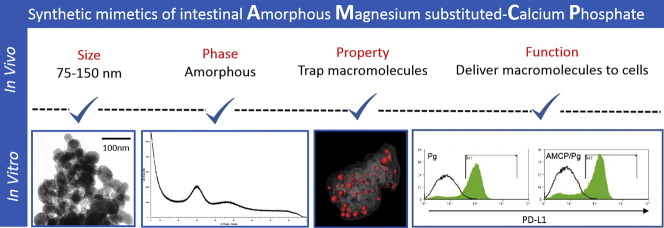

Bio-mineralization is a well conserved physiological phenomenon that occurs in most life forms (bacteria, diatoms, plants, animals and humans)^1^, ^2^, ^3^, ^4^, ^5^ and is usually restricted to specialized tissues (*e.g.* bone, teeth and otoconia) for the formation of calcium-based minerals. In mammals, calcium phosphates are major biominerals and, depending on the tissues involved, may vary in structure from amorphous calcium phosphate (ACP) to fully crystalline forms (*i.e.* apatite). Due to its low toxicity but ready turnover, potential therapeutic roles for ACP are multiple, ranging from bio-materials,^6^ such as in dentistry,^7^ through to drug delivery,^8^, ^9^ such as in vaccination.^10^ However, ACP is notoriously unstable and its *in vivo* ‘lifetime’ is determined not only by environmental factors such as pH, viscosity, ionic strength and temperature^11^, ^12^, ^13^ but also by its own structure, especially the incorporation of other ions or molecules (for *e.g.* fetuin). Boskey & Posner^14^ studied the kinetics of ACP conversion and found that substitution of *Ca* ions in ACP by Mg ions leads to greater stability of the amorphous state, a finding which has since been confirmed by others.^15^, ^16^ Interestingly, we reported that in the human and murine gastrointestinal tract, there is extensive constitutive (*i.e.* natural, physiological) formation of porous amorphous magnesium-substituted calcium phosphate (AMCP) nanoparticles, typically 75-150 nm in diameter.^17^ These luminally-formed AMCP nanominerals are found co-associated with macromolecules such as bacterial peptidoglycan and dietary protein in mucosal antigen presenting cells (APCs).^17^

These findings with endogenous AMCP imply that the particles form in the gut lumen, trapping luminal macromolecules in the process, and then chaperone their cargo to specialized APCs thereby facilitating cross-talk between the lumen and intestinal tissue.^17^ The physiological and biological relevance of this constitutive self-assembly of nanoparticles and their ‘cargo shuttling’ function is currently not fully understood but generation of this knowledge would be greatly facilitated by the synthesis of *in vitro* counterparts such that their properties can be scrutinized.

Here we therefore set out to reproduce the exact form of these entities and, in particular, to test whether the presence of macromolecules (*e.g.*, proteins and bacterial antigens) and ions (*e.g.*, magnesium) would affect the phase, structural properties and stability of the resulting amorphous calcium phosphates. Under carefully controlled conditions, we demonstrated that the synthetic mimetics of the endogenous gastrointestinal nanomineral generated amorphous magnesium-substituted calcium phosphate nanoparticles (synthetic AMCP) of the right size and phase with versatile macromolecule-incorporation properties similar to their *in vivo* counterparts. We next considered synthetic AMCP interactions with immune cells at both the gene and protein level. Particularly, we observed that cargo-containing AMCP nanoparticles were readily taken up by peripheral blood antigen presenting cells, trafficked to lysosomal compartments and delivered their cargo in a ‘silent’ fashion. The synthetic mimetics of the AMCP nanomineral, as described herein, may therefore have valuable properties in drug delivery given that the material does not appear to influence outcomes as may be expected with conventional particle-cargo targeting of cells.^18^

## Methods

### Synthesis of mimetics of endogenous calcium phosphate with organic and inorganic macromolecules

Synthetic AMCP particles were prepared using a modified protocol of Boskey and Posner.^14^ The modification consisted of the addition of extra magnesium and protein (bovine serum albumin, BSA) to enhance phase stability and precipitation in the presence or absence of, mainly, the microbial associated molecular pattern peptidoglycan (Pg) to mimic *in vivo* organic (bacterial) intestinal components. Exact protocols for each of the combinations investigated are given in the supplementary methods (Appendix A).

### Physico-chemical characterization of synthetic AMCP particles

#### Size measurement and structural morphology

Following synthesis, hydrodynamic sizes of the AMCP particles were investigated by nanoparticle tracking analysis while structural morphology was addressed by transmission electron microscopy (TEM). 3D Structural morphology was further investigated using tomography experiments based on high-angle annular dark-field (HAADF) imaging in the scanning transmission electron microscopy (STEM) mode.

#### Elemental composition

Synthetic AMCP particles were dissolved in 5% HNO_3_ (Sigma-Aldrich Company Ltd., Dorset, UK) prior to inductively coupled plasma optical emission spectrometry (ICP-OES) analysis. Samples were analyzed using the JY2000 ICP-OES (Horiba Jobin Yvon Ltd., Stanmore, UK) and *Ca* and P signals were detected at 396.847 nm and 177.440 nm, respectively. Quantification was performed using external standards (Calibration Standard Solutions 1000 ppm, Fisher Scientific UK Ltd., Leicestershire, UK; 0.5-50 ppm). Total *Ca* and P were obtained from particle suspensions collected after synthesis or after dilution in tissue culture medium (TCM). Background correction was performed using the *Ca* and P values obtained from D10 (*i.e.* the TCM) that had been subjected to the same treatment as particle suspensions. The *Ca*/P molar ratio of particle suspensions was calculated as: *Ca*/P = $\left[\frac{C_{\mathrm{Ca}}/{\mathrm{AW}}_{\mathrm{Ca}}}{C_{P}/{\mathrm{AW}}_{P}}\right]$ where C_x_ is the background-corrected concentration of the respective element as measured by ICP-OES and AW_x_ the atomic weight (*i.e.*, 40.08 for *Ca* and 30.97 for P).

#### Determination of zeta potential

Electrophoretic mobility measurements of particle suspensions were performed by phase analysis light scattering on a Zetasizer Nano ZS (Malvern, Worcestershire, UK). Electrophoretic mobility of particles, in an applied field of 8.15 V/cm (effective voltage 49.7 V; electrode spacing 6.1 cm), was then converted into zeta potentials by Dispersion Technology Software 4.20 using Henry's equation and the Smoluchowski approximation for aqueous media. Measurements were taken as technical triplicates, and data are shown as mean of three to five independent experiments.

## Computational modeling

Small precursor calcium phosphate clusters, representative of the early stages of particle nucleation, were constructed and simulated. Posner's cluster,^19^ (Ca_9_(PO_4_)_6_), is considered to be the primary particle of amorphous calcium phosphate and the precursor to the formation of crystalline apatite. This cluster structure was therefore used for the initial model construction, but further modified by the removal of one calcium ion and the addition of two hydrogen ions (for charge compensation) to reflect the experimentally measured mimetic composition as obtained through X-ray microanalysis. Analysis of the clusters' geometric structure and stability under magnesium ion substitution (for calcium ions) in all possible configurations was carried out. The thermodynamic stability was assessed, using formation energy analysis. The formation energy of a single Mg substitution into a calcium phosphate cluster is given by Eq. (1).

(1) $$E_{f}=E_{\mathrm{AMCP}}-\left(E_{\mathrm{ACP}}+\mu_{\mathrm{Mg}}-\mu_{\mathrm{Ca}}\right)$$

where *E*_*AMCP*_ is the energy of the Mg-substituted cluster, *E*_*ACP*_ is the energy of the cluster prior to Mg-substitution, *μ*_*Mg*_ is the chemical potential of magnesium and *μ*_*Ca*_ is the chemical potential of calcium. Chemical potentials for Mg and *Ca* were calculated from the lowest energy sources and sinks, namely magnesium and calcium metal respectively.

Geometry optimization of the AMCP clusters was carried out using the plane-wave density functional theory code, CASTEP.^20^ Convergence testing assigned a kinetic energy cut-off of 430 eV and sampling of the Brillouin zone was carried out at the gamma point^21^ with the clusters placed centrally in a 20Å^3^ simulation cell. Ultrasoft pseudopotentials were employed^22^ along with the generalized gradient approximation (GGA) and PBE exchange-correlation functional.^23^ Convergence tolerances for energy change, maximum displacement and maximum force were set at 1 × 10^−5^ eV atom^−1^, 0.001 Å, and 0.03 eV Å^−1^ respectively. The simulation cell lattice parameters remained constrained throughout. For the substitution of Mg into hydroxyapatite, the lattice parameters were not constrained. Additionally, a maximum stress tolerance of 0.05 GPa and a 3 × 3 × 3 *k*-point grid for sampling of the Brillouin zone were employed.

### Macromolecule trapping properties of synthetic AMCP

AMCP-trapping of macromolecules was assessed by determining the content of BSA and Pg from re-dissolved nanoparticles using protein and periodic acid Schiff assays, while incorporation was demonstrated by STEM tomographic reconstruction of synthetic AMCP containing electron dense material, namely ultra-fine iron (III) oxohydroxide.^24^ Detailed methodology can be found in Appendix A, supplementary Methods.

### Biological properties of synthetic AMCP

Studies with primary human cells and experimental protocols were approved by the research ethics committees of Cambridge, UK (reference 03/296) and carried out in accordance with approved guidelines. The biological properties of ACMP nanoparticles were examined in peripheral blood mononuclear cells, enriched monocytes and/or THP-1 as stated. AMCP nanoparticle uptake and lysosomal co-localization were assessed by flow imaging techniques while AMCP-induced biological responses were investigated by looking at ‘toxicity’, ‘inflammasome activation’, IL-1β responses and whole genome transcriptomic analyses. Full methods are given in Appendix A, supplementary Methods.

### Statistics

Significant differences in experiments looking at the inflammasome activation were assessed by a two way ANOVA and Bonferroni's multiple comparisons test while experiments looking at cellular uptake, cell viability and surface PD-L1 expression were evaluated using 2 tailed paired *t* tests. Significance was set as *P* < 0.05. For transcriptomic studies, significant differences in expression were assessed using paired Intensity-Based Moderated T-statistic (IBMT).^25^ Genes were defined as significantly changed when the *P* value was <0.01.

## Results

### Physico-chemical and structural properties

**Figure 1:**
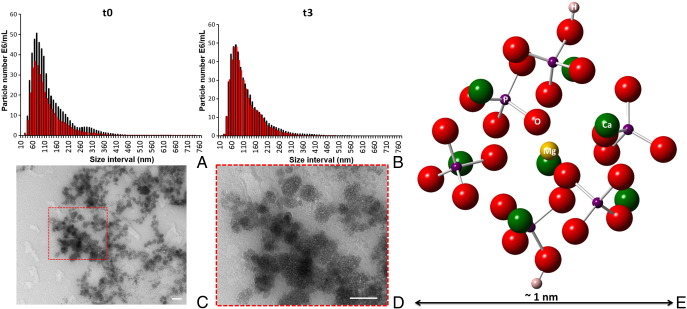
Sizing and TEM morphology of synthetic AMCP nanomineral from cell culture medium: (**A-B**) Nanoparticle tracking analyses of synthetic AMCP particles that were prepared in the presence of bovine serum albumin (BSA) with (red columns) and without bacterial peptidoglycan (sPg; black columns), either (**A**) in cell culture medium (37 °C) immediately after suspension or (**B**) following 3 h incubation (37 °C) in cell culture medium. Size distributions are shown as average of three independent experiments and are displayed as cumulative particle number (E6/ml) per size interval (*i.e.* every 10 nm). Supplementary Table 1 provides additional data. (**C-D**) Morphologic characterization of the above particles by TEM after 1 h incubation of the particles in cell culture medium at 37 °C. Scale bars: 100 nm. Detailed STEM analysis of the precipitated and washed powder prior to addition to cell culture medium is shown in Figure 2. (**E**) Energy optimized structure of Mg-substituted calcium phosphate cluster, which represents a precursor to the larger particles. The central Mg substitution is in the most favorable position as calculated by Density Functional Theory. The composition of the cluster reflects analytical results of the larger particles by X-ray microanalysis. Hydrogen was included for charge compensation. Calcium is shown in green, oxygen in red, phosphorus in purple, magnesium in yellow and hydrogen in pink.

Following a screen of numerous candidate synthetic methods, we found that co-precipitation of a model protein (bovine serum albumin; BSA) at 500 μg/ml with 17.5 mM Ca^2+^, 19.5 mM phosphate and 3.6 mM Mg^2+^ in pH 8 aqueous buffer enabled the recovery of a precipitate that, when washed and re-suspended in tissue culture medium, consisted of disperse AMCP nanoparticles with a hydrodynamic size distribution (D10-D90) from ~50 to 215 nm, a D50 of ~100 nm and a peak size at about 70 nm (Figure 1 and Table S1). Once re-suspended in tissue culture medium, the particle size, measured by nanoparticle tracking analysis, remained stable for at least 3 h (Figure 1 and Table 1). The additional presence of peptidoglycan (50 μg) during co-precipitation did not impact these findings (Figure 1 and Table 1).

**Figure 2:**
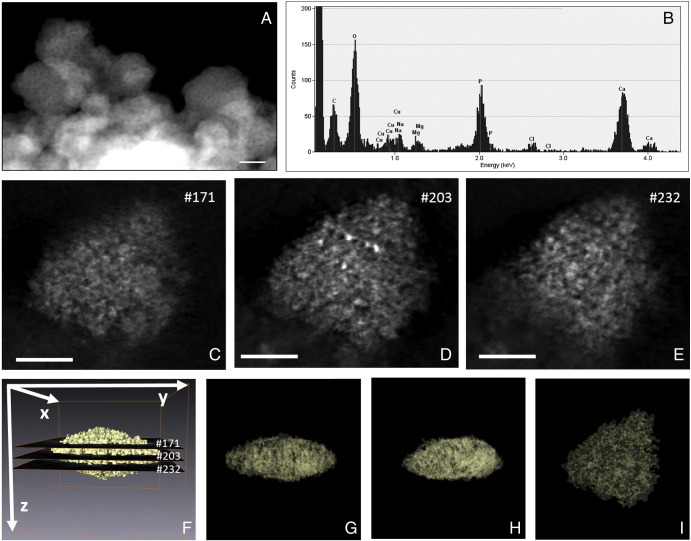
STEM characterization of synthetic AMCP nanoparticles: (**A**) High angle annular dark-field STEM image of synthetic AMCP particle clusters (Scale bar 20 nm) and (**B**) example elemental composition by energy dispersive X-ray microanalysis. (**C-E**) A series of orthoslices of a synthetic AMCP particle (Scale bar 20 nm) revealing a detailed inner structure (**F-I**) from the reconstructed volume (**F**) by means of several orthoslices through the XY plan and (**G-I**) transparency views through the YZ, XZ and XY orientations, respectively.

Morphologically, agglomerates of non-facetted, micro-porous particles were evident and these were shown to be amorphous by TEM, whether pre-incubated or not in tissue culture medium (Figure 1, *C-D* and Figure 2, *A*). To ensure that the optimal synthetic composition had been attained, first principles Density Functional Theory (DFT) modeling of an amorphous calcium phosphate cluster model (Figure 1, *E*) based on the anticipated primary particle, which is termed ‘Posner's Cluster’,^19^ was undertaken. Prior to undergoing geometry optimization, the initial ‘Posner's Cluster’ composition was modified to reflect the X-ray microanalysis results of the larger particles presented in Figure 2, *B*, with a reduction in *Ca* ion concentration and the addition of two H ions for charge compensation. Simulations showed that Mg was most favorable in the central cation position of the cluster. This optimized model was then used to interrogate the stability of AMCP with differing Mg (substituting for *Ca*) concentrations. As previously reported for bulk phase amorphous calcium phosphate 7, 11, 14 the presence of both Mg^2+^ ions and protein stabilized the amorphous nanoparticles and retarded/prevented conversion to crystalline calcium phosphate (Figure S1). However, formation energy analysis, which quantitatively assesses the thermodynamic stability of an elemental or molecular substitution in a structure, clearly showed that concentrations of Mg greater than that of our mimetic were thermodynamically unfavorable and that the optimal Mg concentration (which had a negative formation energy of −0.463 eV) had already been attained, Table 2. Furthermore, Mg substitution into the crystalline calcium phosphate phase hydroxyapatite was shown to be energetically unstable, with a positive formation energy of +1.747 eV. This demonstrated that at our synthetic composition, the amorphous phase would retain its integrity and effectively prevent crystallization.

**Figure 3:**
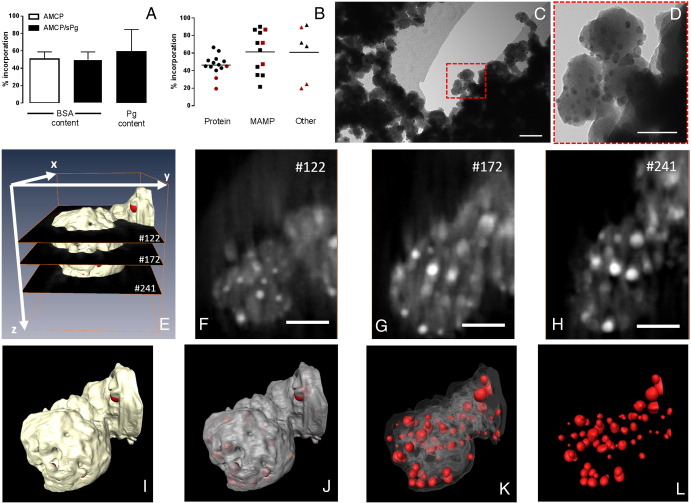
Trapping of macromolecules by the synthetic AMCP nanomineral: (**A**) Percentage incorporation, from the original solution, of protein (BSA; left hand-side and middle column) and bacterial peptidoglycan (sPg, right hand-side column) in synthetic AMCP prepared in the presence of BSA with (black columns) and without peptidoglycan (white column), and as determined by the Bradford protein and periodic acid Schiff assay, respectively (n = 5-6 experiments ± SD). (**B**) Overall percentage incorporation of protein (black circles are BSA and red circles are avidin), bacterial fragments (black squares are sPg and red squares are LPS) and other macromolecules (black triangles are soluble starch and red squares are retinoic acid) in synthetic AMCP. (**C-D**) TEM of synthetic AMCP nanoparticles prepared in the presence of iron oxide nanoparticles showing fine nano-iron particles densely incorporated into the AMCP structure. Scale bars 100 nm and 50 nm respectively. (**E-H**) STEM-tomography data of synthetic AMCP, containing fine nano-iron particles, from the reconstructed volume by means of several orthoslices through the XY plane and (**F-H**) showing individual slices and finally (**I-L**) the 3D reconstruction in differing views. The iron oxide nanoparticles are shown in red in I-L.

Consistent with our prior *in vivo* findings,^17^ analysis by high angle annular dark field scanning transmission electron microscopy (HAADF-STEM; Figure 2, *C-E*), and then 3D-reconstruction of protein-loaded synthetic AMCP clusters (Figure 2, *F-I*) confirmed the porous nature of the particles (pore average size of 1-3 nm). Under the synthetic conditions described above ~50% of the 500 μg/ml BSA protein was trapped by AMCP whether peptidoglycan was present or not (Figure 3, *A*). About 60% of the 50 μg/ml peptidoglycan was also trapped (Figure 3, *A*) such that, overall, the AMCP construct contained 10% protein and 1% bacterial peptidoglycan by weight (Table 1). The synthetic AMCP nanoparticles were versatile and non-specific in the trapping of organic matter since other proteins (*e.g.*, avidin) and molecules such as bacterial lipopolysaccharide (LPS), soluble starch (a complex carbohydrate) and retinoic acid (vitamin A-derived compound) were all efficiently incorporated, from 20% to 90% of their original solution concentration (Figure 3, *B*). To facilitate later work that required the use of fluorescent cargo for cellular experiments, we also show that fluorescently-labeled macromolecules (Alexa Fluor 555 BSA and Texas Red OVA) were trapped by AMCP (Figure S2, *A*). It is noteworthy that the presence of Mg not only provided stability of the particle's phase but it also promoted a greater association with proteins than regular amorphous calcium phosphate free of Mg (*i.e.* ACP; Figure S2, *B-C*).

To support the idea that the internal porosity observed by STEM is a result of the AMCP mineral templating around much smaller macromolecular components during synthesis, we undertook further studies with an electron dense macromolecule to enable visualization by electron microscopy. Synthetic AMCP nanoparticles were therefore prepared in the presence of ultra-fine iron (III) oxohydroxide nanoparticles^24^ (5-10 nm diameter). Electron microscopy imaging (Figure 3, *C-D*), tomographic reconstruction (Figure 3, *E-H*), models with increasing transparency views (Figure 3, *I-L*) all confirmed that the fine nano-iron particles were densely incorporated *within* the synthetic AMCP.

### Biological properties

We next sought to investigate, *in vitro*, whether synthetic AMCP nanoparticles would modulate cellular responses and we took a step-wise approach where AMCP uptake, cargo delivery, toxicity, inflammasome activation and gene expression modulation were investigated.

**Figure 4:**
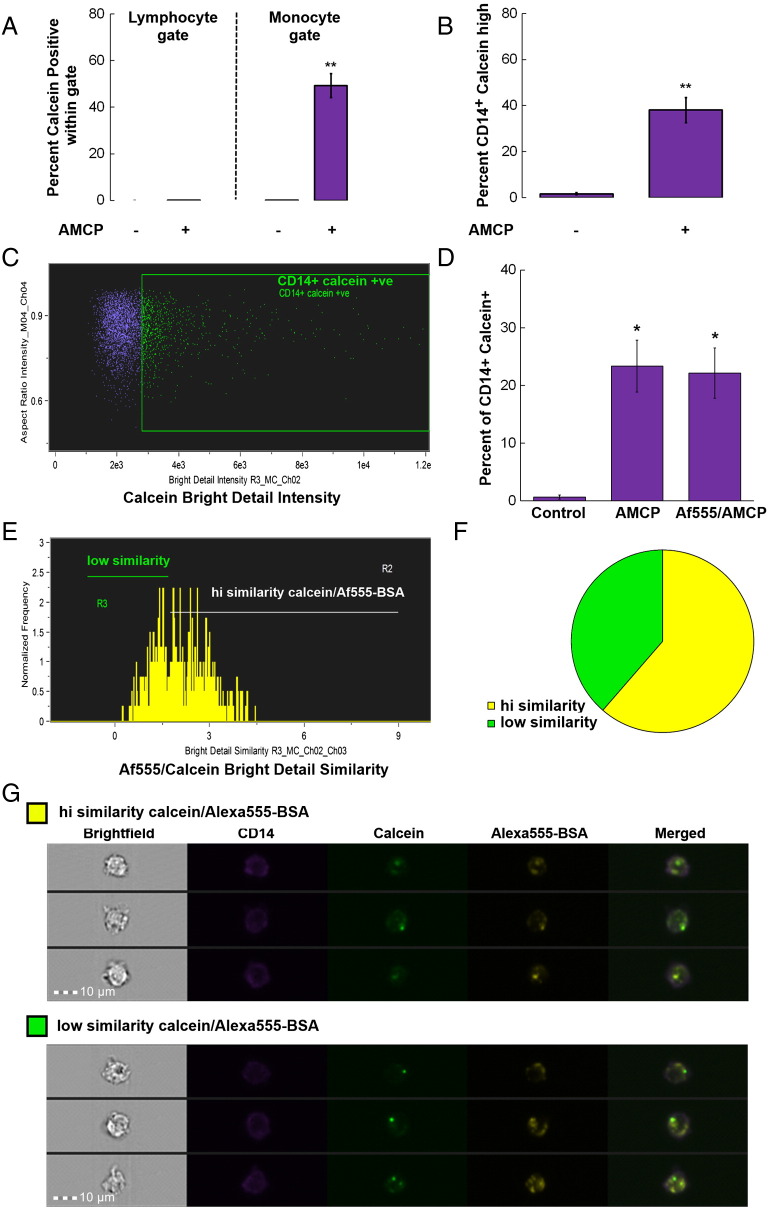
*In vitro* uptake of synthetic AMCP nanoparticles and delivery of cargo into cells: (**A**) Flow cytometric quantitation of cells that have taken up synthetic calcein-labeled AMCP within the lymphocyte and monocyte gates (n = 3 ± SD). (**B**) Image Stream analyses of PBMC that were incubated with synthetic calcein-labeled AMCP displaying the monocyte population that have taken up AMCP (n = 4 ± SD). **P = 0.01 *versus* vehicle. (**C**). Example of Image Stream dot plot from one donor showing gated CD14^+^ cells positive for the AMCP label calcein, and (**D**) graph showing the average percentage of CD14^+^ cells positive for calcein after 3 h incubation with AMCP particles synthesized with or without Af555 labeled BSA. (**E**) Example histogram plot of calcein/Af555-BSA similarity scores of double positive cells for 1 subject after 3 h incubation, showing high and low similarity regions. (**F**) Pie Chart visualizing percentage of CD14^+^ (calcein/Af555 double positive) cells falling within high (yellow) and low (green) similarity regions: data shown are the average of experiments performed on cells from 3 subjects. (**G**). Example cell images of calcein/Af555 double positive CD14 cells falling within the high and low similarity gate.

By means of calcein staining and flow imaging analyses we confirmed that synthetic AMCP nanoparticles were efficiently and selectively taken up by APCs in culture, namely CD14^+^ monocytes from a mixed cell culture of peripheral blood mononuclear cells (PBMC; Figure 4, *A-B*, *P* = 0.01). To then examine protein carriage by AMCP and delivery into cells, we used BSA fluorescently tagged with Alexa Fluor 555 to track the incidence of protein within cells of the monocyte lineage in conjunction with fluorescently labeled AMCP. Twenty-two percent (±7; SD) of monocytes within PBMC were positive for AMCP and identified as calcein positive CD14^+^ cells. The addition of fluorescent protein during AMCP synthesis did not alter the percentage of cells displaying calcein positivity (Figure 4, *C-D*). Of this AMCP (calcein) positive fraction of CD14^+^ cells, over half were also Alexa Fluor 555 bright indicating the presence of BSA protein in addition to AMCP (data not shown). These double positive cells were then further assessed for the spatial similarity of the 2 fluorescent markers. This analysis suggested that AMCP and protein fluorescence displayed a high degree of similarity in 60% (± 7; SD) of the CD14^+^ cells positive for both fluorescent markers (Figure 4, *E-F*). Examples of high and low similarity images are shown in Figure 4, *G*. Overall these data not only corroborate the association of AMCP with macromolecules but also demonstrate carriage and delivery of protein into CD14^+^ cells by AMCP as indicated by the co-incidence of the respective fluorescent markers within cells.

**Figure 5:**
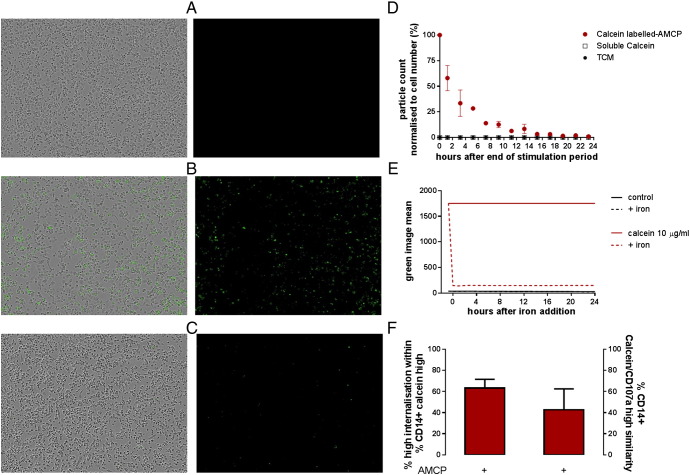
Uptake, lysosomal *delivery* and dissolution of synthetic AMCP nanoparticles. PMA-differentiated THP-1 macrophages were incubated with calcein-labeled AMCP for 3 h and then washed to remove residual AMCP particles. Images were then acquired every 2 h by the Incucyte™. (**A-C**) Representative images from the green channel (right hand-side) and overlay images of phase contrast and green fluorescence (left hand-side) that were acquired one hour-post stimulation with (**A**) TCM, (**B**) AMCP and (**C**) 11 h-post stimulation with AMCP (10× magnification). (**D**) Particle count, as quantified by the IncucyteZOOM software, was normalized against cell confluence, and is expressed as percent of values obtained in the first scan. X-axis indicates monitoring time by live cell imaging post-stimulation, *i.e.*, 0 h represents the first scan (*i.e.* straight after 3 h stimulation with calcein-labeled AMCP). Data are presented as mean ± SD (n = 5, obtained from two independent experiments performed in technical replicates). (**E**) Mean green fluorescence intensity of soluble calcein added at indicated concentrations to tissue culture medium (acellular environment) and measured by the Incucyte™. Dye quenching was induced by the addition of 1 mM iron as iron hydroxide adipate tartrate. Data are shown as connecting curves for the mean of technical triplicates from one experiment. (**F**) Image Stream summary of internalized synthetic calcein-labeled AMCP in peripheral blood monocytes (left column) that were concomitantly positive for the lysosomal marker CD107 (Right column; n = 4 ± SD).

We then used adherent macrophages (differentiated THP-1) to study AMCP dissolution in real time using live imaging (see supplementary methods). This showed avid uptake of calcein-labeled AMCP (Figure 5, *B*) while no fluorescence could be observed after incubation with calcein alone (Figure 5, *A*). In cells, the count of discrete, punctate green-fluorescent objects decreased gradually over time (Figure 5, *C*) and became undetectable 18 h post-stimulation hence confirming that synthetic AMCP nanoparticles were dissolved/digested (Figure 5, *D*; effect of time *P* < 0.0001). Loss of calcein fluorescence was not observed when calcein solutions were incubated alone (Figure 5, *E*), consistent with intracellular dissolution of ‘particulate calcein’ rather than dye quenching over time (Figure 5, *E*). Indeed, since AMCP nanoparticles were transported to CD107^+^ (LAMP1^+ve^) lysosomal compartments (Figure 5, *F*) of peripheral blood monocytes, the time-dependent decline of the fluorescence is consistent with intracellular particle digestion in the acidic environment of endo-lysosomes.^26^, ^27^, ^28^

Next we investigated APC responses to protein-loaded nanomineral AMCP with and without peptidoglycan.

**Figure 6:**
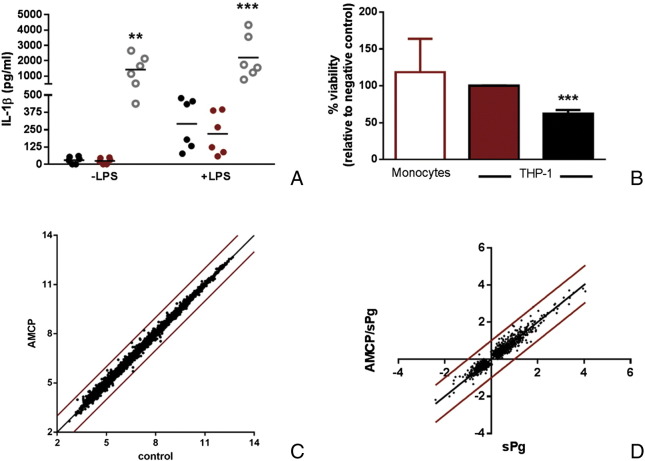
*In vitro* gene and cellular responses to AMCP nanoparticles: (**A**) Lack of inflammasome activation by synthetic AMCP as measured by IL-1β secretion following LPS pre-stimulation (10 ng/ml) and subsequent challenge with vehicle (TCM; black circle) or synthetic AMCP (red circle) or with bacterial peptidoglycan (gray circle). Positive control crude peptidoglycan shown in open gray circles. **P < 0.01 and ***P < 0.001 *versus* vehicle (n = 6). (**B**) Cell viability was assessed by flow cytometry in monocytes (white column, n = 5 ± SD) and by the LDH assay in THP1 cells (red column, duplicates). ***P = 0.0003 *versus* AMCP (red column). (**C**) Average log_2_ expression values of genes, after 3 h exposure to synthetic AMCP, correlated against those of vehicle control treatment (n = 7). (**D**) Average log_2_ fold changes of genes significantly affected (*P* < 0.01, n = 7) by 3 h treatment with synthetic AMCP/sPg nanoparticles or with sPg alone (both *versus* vehicle control) and plotted against each other. Theoretical line of perfect correlation (black line) and borders corresponding to twofold up- and down-regulation (red lines) were overlaid on the data in both c and d.

Canonical activation of the inflammasome and IL-1β production usually require a two-signal process^29^ but may also arise from lysosomal disruption preceding cell death which can be induced, for example, by the gorging of calcium phosphate nanoparticles.^30^ To demonstrate the role of synthetic AMCP in inflammasome activation, blood-derived mononuclear cells were first subjected to vehicle or LPS pre-stimulation to induce pro-IL1β synthesis, and IL-1β production was measured 21 h after a 3 h-challenge with synthetic AMCP. Under conditions where artifactual activation of the inflammasome is avoided,^30^ it was evident that synthetic AMCP, in contrast to crude peptidoglycan (Figure 6, *A*; *P* < 0.01) and to some calcium phosphate crystals,^31^, ^32^ did not activate the inflammasome platform as the levels of secreted IL-1β were similar to those observed for the control (*i.e.* vehicle alone; Figure 6, *A*). Moreover, cellular uptake of synthetic AMCP was innocuous even under prolonged experimental conditions where particle gorging may ensue^30^ (*i.e.*, following 24 h exposure in culture) as cell viability was not affected in primary cells or THP-1 cells (*P* = 0.0003 *versus* apatitic calcium phosphate-challenged cells; Figure 6, *B*).

Having observed neither toxicity nor inflammasome activation by the AMCP nanomineral under the tested conditions, we next considered what cellular responses *did* ensue. We looked at responses across the whole genome and, remarkably, found that cells challenged with protein-loaded synthetic AMCP nanoparticles displayed exactly the same transcriptomic ‘signature’ to that of unchallenged cells (Figure 6, *C*). Since peptidoglycan is the inferred signaling molecule in the nanomineral-antigen-peptidoglycan pathway, we next sought how synthetic AMCP nanoparticles, loaded with both protein *and* peptidoglycan, affected peptidoglycan ‘signaling’. Masking (*i.e.*, down regulation of the cell's sensitivity to peptidoglycan) and enhancing recognition were both possibilities so, again, we used whole genome transcriptomics as a read-out. Whether delivered in soluble form or as cargo within the synthetic AMCP nanoparticles, gene regulation in response to ultra-pure *E.Coli* peptidoglycan was *precisely* the same (*i.e.*, no gene regulation differed by more than the ‘cut-off’ of 2-fold; Figure 6, *D*).

**Figure 7:**
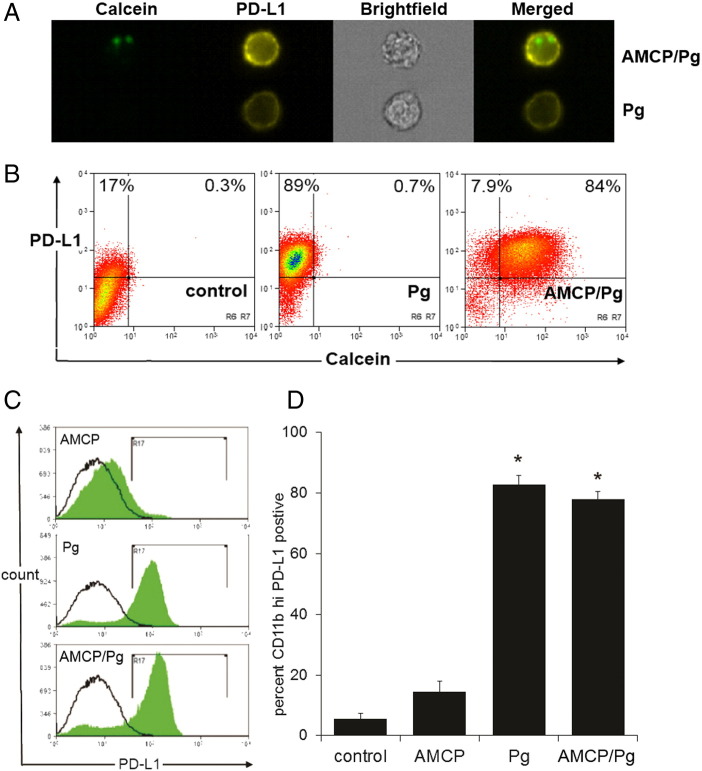
Peptidoglycan cargo of AMCP nanomineral upregulates cellular PD-L1 expression: (**A**) Cell surface PD-L1 is upregulated in antigen presenting cells by peptidoglycan (Pg) and by Pg delivered in synthetic nanomineral particles (AMCP/Pg) that were visualized by pre-labeling with calcein. (**B**) Example flow cytometric plots of CD11b^+^ pre-gated cells showing dual calcein/PD-L1 positivity upon stimulation with vehicle (control), peptidoglycan (Pg) or calcein-tagged Pg-bearing synthetic nanomineral particles (AMCP/Pg). (**C**) Representative histograms showing PD-L1 positivity of CD11b^+^ gated cells following exposure to protein-bearing synthetic nanomineral particles (AMCP), peptidoglycan (Pg) or Pg-bearing nanomineral particles (AMCP/Pg). (**D**) Mean ± SEM PD-L1^+^ CD11b^+^ cells within the monocyte gate of peripheral blood mononuclear cell populations derived from 7 healthy donors following exposure to protein-bearing synthetic nanomineral particles (AMCP), peptidoglycan (Pg) or Pg-bearing synthetic nanomineral particles (AMCP/Pg). * Asterisked data are not significantly different while *P* < 10^−6^ for asterisked *versus* non-asterisked data.

These findings also showed therefore that, *PD-L1*, the likely target for peptidoglycan signaling in the AMCP nanomineral pathway *in vivo*,^17^ was equally upregulated whether peptidoglycan from *E. coli* was delivered alone or as cargo (Figure S3). Given that peptidoglycan also derives from Gram^+^ bacteria, we finally confirmed that peptidoglycan from Gram^+^*S. aureus*, alone or delivered by the AMCP nanomineral, similarly imprinted PD-L1 expression on APCs in *in vitro* cell culture (Figure 7).

**Figure 8:**
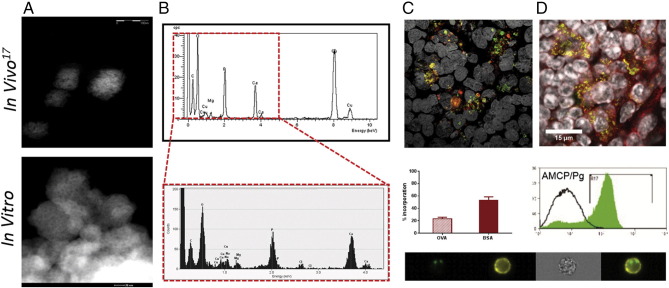
Comparisons of *in vitro* synthetic AMCP to the *in vivo* AMCP nanomineral. The upper panel displays the *in vivo* characteristics of AMCP and has been reproduced with permission from Nature Nanotechnology (adapted from Powell JJ et al^17^). The lower panel is a summary of the characteristics of *in vitro* generated AMCP in this work. (**A**) High angle annular dark-field STEM image of AMCP particle clusters. (**B**) Elemental composition by energy dispersive X-ray microanalysis. (**C-D**) Upper panels: (**C**) *In vivo* confocal micrographs showing a 3D stack, with Huygens MLE deconvolution, visualized with calcein staining for the endogenous nanomineral (green) and with antibody staining for ovalbumin (red) and (**D**) PD-L1 (red) expression in calcein positive (green) cells. (**C-D**) The lower panels displays the *in vitro* equivalence and shows the percentage of fluorescent protein (red column for BSA555 and red dashed column for Texas Red OVA) present within AMCP, the uptake of synthetic AMCP (green) into PD-L1^+^ cells (yellow) by Image Stream and the resulting PD-L1 up-regulation (green histogram) by Flow cytometry.

Together, our data demonstrate that *in vitro* synthetic AMCP nanoparticles mimic their *in vivo* endogenous counterparts in terms of size (Figure 8, *A*), elemental composition (Figure 8, *B*), and in the trapping of macromolecules (cargo) allowing effective delivery to cells (Figure 8, *C*). Moreover we show that the *in vivo* reported biological function of PD-L1 up-regulation, driven by the peptidoglycan cargo,^17^, ^33^ is conserved *in vitro* (Figure 8, *D*).

## Discussion

Constitutive formation of AMCP nanoparticles, typically 75-150 nm in diameter, is a normal physiological and conserved process that occurs in the gastrointestinal tract of different mammalian species.^17^ During its formation, the AMCP nanomineral co-associates with macromolecules that are present in the lumen and, like other non-biological particles of similar sizes,^34^ efficiently crosses the epithelial barrier *via* M cells of the Peyer's patches.^17^ The cargo-containing nanomineral transiently accumulates in APCs underlying the epithelium where mineral digestion and release of the cargo occur.^17^ This process likely promotes immuno-surveillance of luminal antigens. In this work, we aimed to produce synthetic forms of these particles with a view to start trying to establish some of their biological relevance in *in vitro* investigations.

Our findings demonstrate that synthetic AMCP nanoparticles act as effective but entirely silent delivery platforms both in their own right and also in terms of cellular responses to peptidoglycan. For two reasons these findings are surprising. Firstly, while failure to activate the inflammasome by apatitic calcium phosphate nanoparticles has been reported recently by us, a lack of cell death following gorging (*i.e.* up to 24 h exposure to synthetic AMCP nanomineral) was unexpected based upon these prior studies.^30^ It is possible that the cargo contained within the AMCP confers protection against cell death by slowing down lysosomal dissolution of the nanoparticles, as recently demonstrated for fetuin and calcium phosphate in human vascular smooth muscle cells,^35^ ensuring cell safety of the entire conjugate. Secondly, nanoparticles, including nano forms of calcium phosphates, have been widely used as adjuvants to enhance immunogenicity to protein antigens and microbial associated molecular patterns,^36^, ^37^, ^38^ again unlike the role of the particles in this study. The anticipated *in vivo* function of endogenously-formed AMCP may require this inert (silent-delivery) nature of the cargo-bearing particles because the default setting for gastrointestinal immune cells is one of hyporesponsiveness and tolerance towards non-self antigens.^39^, ^40^ While the AMCP is inert, its cargo (notably peptidoglycan) appears to actively promote tolerance in the normal gut by up-regulating the co-regulatory molecule, PD-L1, on APCs that are recipients of endogenous cargo-bearing AMCP.^17^, ^33^ However, this is not the case in Crohn's disease.^41^ We have recently shown that APCs of the AMCP pathway display a selective failure in expression of PD-L1 in this disorder^41^ and hence hypothesized that this may lead to a loss of tolerance towards luminal contents—a hallmark of the disease.^42^ It is therefore likely that, under normal circumstances, the mammalian gut immune system has evolved to use an endogenous carrier of luminal macromolecules that can (a) trap luminal proteins and microbial associated molecular patterns effectively, (b) allow transport to APCs while protecting the cargo from enzymatic degradation *en route* and, pertinent to the findings here, (c) not promote cellular reactivity in a naturally hyporesponsive environment.

In summary, the synthesis of AMCP has been inspired by its presence in nature and it may be a rather special nanomaterial that does not, acutely, promote cell activity following its uptake nor shield the cell signaling of its cargo. Further work should now consider AMCP for the delivery of immunogenic protein (with and without bacterial fragments) and see how this might influence cell mediated immunity. This would further inform on function and perhaps provide a novel therapeutic nano-carrier, especially for impacting tolerance in the gut.

## Figures and Tables

**Table 1:** Physico-chemical characteristics of synthetic AMCP nanoparticles after synthesis and in cell culture conditions.

		Organic content (μg/mg; n = 5-9 ± SD)	*Ca*/P (n = 3 ± SD)	ζ potential (n = 3-5 ± SD)
Synthesis	AMCP	BSA 103.92 ± 17.01	1.29 ± 0.012	-13.22 ± 0.76
	AMCP/sPg	BSA 86.74 ± 15.37 sPg 10.23 ± 4.46	1.30 ± 0.017	-13.06 ± 0.31
Cell culture conditions t0	AMCP	N/A	1.23 ± 0.026	-12.14 ± 0.49
	AMCP/sPg	N/A	1.29 ± 0.056	-12.97 ± 1.49
Cell culture conditions t3	AMCP	N/A	1.29 ± 0.01	N/A
	AMCP/sPg	N/A	1.31 ± 0.06	N/A

**Table 2:** Formation energies of magnesium substitution (substituting for calcium ions) into an energy optimized amorphous calcium phosphate model, based on a modified Posner’s Cluster.

Structure	Particle Formula	Formation Energy (eV)
Unsubstituted primary cluster	Ca_8_(PO_4_)_6_H_2_	–
	Ca_7_Mg(PO_4_)_6_H_2_	-0.463
	Ca_6_Mg_2_(PO_4_)_6_H_2_	+1.644
	Ca_5_Mg_3_(PO_4_)_6_H_2_	+2.705
Mg-substituted clusters	Ca_4_Mg_4_(PO_4_)_6_H_2_	+4.244
	Ca_3_Mg_5_(PO_4_)_6_H_2_	+7.412
	Ca_2_Mg_6_(PO_4_)_6_H_2_	+7.902
	CaMg_7_(PO_4_)_6_H_2_	+10.971
Lowest energy Mg-substituted HA	HA - Mg	+1.747

Substitution of a single Mg ion into a unit cell of hydroxyapatite (HA) is also presented. Positive formation energies indicate an unfavorable/unstable structure and negative formation energies a favorable/stable structure.
