# Supplementary material for: Synthetic mimetics of the endogenous gastrointestinal nanomineral: Silent constructs that trap macromolecules for intracellular delivery
Source: Nanomedicine. 2017 Feb;13(2):619–30. doi: 10.1016/j.nano.2016.07.008 (PMC5339085; doi:10.1016/j.nano.2016.07.008)
Supplement: Supplementary file 1 — Supplementary material. [file mmc1.docx]

**Synthetic mimetics of the endogenous gastrointestinal nanomineral: silent constructs trap macromolecules for intracellular delivery.**

Laetitia C Pele, Carolin T Haas, Rachel E Hewitt, Jack Robertson, Jeremy Skepper, Andy Brown, Juan Carlos Hernandez-Garrido, Paul A Midgley, Nuno Faria, Helen Chappell & Jonathan J Powell.

**Supplementary Methods:**

**Synthesis of mimetics of endogenous calcium phosphate with organic and inorganic macromolecules**

Synthetic AMCP particles were prepared using a modified protocol of Boskey and Posner ^1^. The modification consisted of the addition of extra magnesium and protein (bovine serum albumin, BSA) to enhance phase stability, and the presence or absence of, mainly, the microbial associated molecular pattern peptidoglycan (Pg), to mimic *in vivo* organic (bacterial) intestinal components. Exact protocols are given below for each of the combinations investigated.

Synthesis of empty particles or “AMCP”: BSA (Sigma-Aldrich Company Ltd., Dorset, UK) or BSA 555 (Life Technologies) or Texas Red OVA (Life Technologies), was dissolved in a Ca/Mg solution (containing 35 mM CaCl_2_ (Sigma-Aldrich), 7.2 mM MgCl_2_ (Sigma-Aldrich) in 0.15 M TRIS buffer (Sigma-Aldrich) and adjusted to pH 8 with hydrochloric acid) at 1 mg/ml. To form calcium phosphate particles, the resulting Ca/Mg/Protein solution was then mixed with an equal volume of phosphate (PO_4_) solution (containing 39 mM (NH_4_)HPO_4_ (Sigma-Aldrich) in 0.15 M TRIS buffer, adjusted to pH 8 with hydrochloric acid).

Synthesis of “ACP” containing BSA555 or Texas red OVA: BSA 555 (Life Technologies) or Texas Red OVA (Life Technologies), was dissolved in a Ca solution (containing 35 mM CaCl_2_ (Sigma-Aldrich) in 0.15 M TRIS buffer (Sigma-Aldrich) and adjusted to pH 8 with hydrochloric acid) at 1 mg/ml. To form calcium phosphate particles, the resulting Ca/Protein solution was then mixed with an equal volume of phosphate (PO_4_) solution (containing 39 mM (NH_4_)HPO_4_ (Sigma-Aldrich) in 0.15 M TRIS buffer, adjusted to pH 8 with hydrochloric acid).

Synthesis of Pg-containing particles or “AMCP/Pg”: In this instance, Ca/Mg/BSA solution was mixed with either soluble Pg (from *Escherichia coli*; Source BioScience plc, Nottingham, UK) or crude Pg (*Staphylococcus Aureus*, Sigma). Resulting solutions were then mixed with an equal volume of PO_4_ solution to allow particle formation and to yield final Pg concentrations of 50 μg/ml.

Synthesis of iron oxide-containing particles or “AMCP/Fe”: Here, Ca/Mg/BSA was mixed with a suspension of iron oxide nanoparticles at 100 μg/ml solution (as prepared and reported in Powell JJ *et al*, 2014) ^2^. The resulting solution was then added to an equal volume of PO_4_ solution to allow particle formation.

Synthesis of LPS-containing particles or “AMCP/LPS”: Ca/Mg/BSA solution was mixed with 100 μg/ml LPS (from *Escherichia coli*, Sigma). The resulting solution was then mixed with an equal volume of PO_4_ solution to allow particle formation.

Synthesis of soluble starch-containing particles or “AMCP/sStarch”: Ca/Mg/BSA solution was mixed with 1 mg/ml soluble starch (Sigma). The resulting solution was then mixed with an equal volume of PO_4_ solution to allow particle formation.

Synthesis of avidin-containing particles or “AMCP/Avidin”: Avidin (Sigma) was dissolved in a Ca/Mg solution (containing 35 mM CaCl_2_ (Sigma-Aldrich), 7.2 mM MgCl_2_ (Sigma-Aldrich) in 0.15 M TRIS buffer (Sigma-Aldrich) and adjusted to pH 8 with hydrochloric acid) at 1 mg/ml. The resulting Ca/Mg/Avidin solution was then mixed with an equal volume of PO_4_ solution.

Synthesis of calcein-labelled AMCP: Ca/Mg/BSA solution was mixed with an equal volume of phosphate (PO_4_) solution. Immediately prior to mixing the two solutions, fluorescent calcein stock solution (10 mg/ml in DMSO; Sigma C0875) was added to the phosphate solution at a concentration of 1 μl per ml mixed solution. The resulting suspension was protected from light thereon.

All particle suspensions were then rotated for one hour at room temperature, washed twice in H_2_O (pH 10) (1500 rpm, 5 min) and finally re-suspended in serum-containing tissue culture medium (TCM), namely D10 or R10 (Dulbecco’s Modified Eagle’s Medium and RPMI-1640 (Sigma-Aldrich), supplemented with 10 % Fetal Calf Serum (FCS; PAA Laboratories Ltd, Somerset, UK), 2 mM L-glutamine (Sigma-Aldrich), 100 U/ml penicillin (Sigma-Aldrich) and 100 μg/ml streptomycin (Sigma-Aldrich)). For downstream applications and where appropriate synthetic AMCP particles were then diluted (1:9) with TCM and incubated for up to 24 hours at 37 °C/95% air/5 % CO_2_, to simulate conditions of cell stimulation.

**Physico-chemical characterisation of synthetic AMCP particles**

Size measurement and structural morphology

Nanoparticle tracking analysis was performed on a Nanosight NS500 (Nanosight, Amesbury, UK) using NTA2.2 Analytical Software. For each experiment samples were measured in technical triplicates (90 sec each) and results averaged after export into Microsoft Excel 2010. Data are shown as mean of three independent experiments. Mode values were obtained through area-under-curve analysis in GraphPad Prism 6.03 while average size and percentiles were determined in Microsoft Excel. For the latter, data were transformed into cumulative percentage and exact D10, D50 and D90 values calculated with linear interpolation using the two data points above and below the 10^th^, 50^th^ and 90^th^ percentile, respectively. For structural morphology, drops of AMCP particle suspensions were placed on a 400 mesh copper grid and analysed by transmission electron microscopy (TEM).

Synthetic AMCP particles were also encapsulated in gelatine, embedded in the epoxy resin ‘Quetol 651’ and sections cut to 70 nm thicknesses. Tomography experiments based on high-angle annular dark-field (HAADF) imaging in the scanning transmission electron microscopy (STEM) mode were performed at 200 kV on a FEI Tecnai F20 FEG-TEM tilting the sample from -42° to +70° at 2° intervals about a single axis using a Fischione 2020 ultra-high-tilt tomography holder. Using the Inspect 3D software, tilt series were aligned by cross-correlation and reconstructed using the SIRT algorithm (with 20 iterations). 3D surface rendering was carried out using the AMIRA Visualisation Package (version 4.1.1, Visage Imaging, Berlin, Germany). In total 1149 slices from the SIRT reconstruction were used for volume rendering of the acquired greyscale image stacks. The SIRT stack was linked to the Amira 4.1 Voltex tool, as well as to a Colormap tool to allow manual adjustment of both colours and transparency functions. The settings used (maximum intensity projection, alpha, 3D texture mode) were applied across the entire number of slices to produce the final rendered surface. This final high image quality non-segmented reconstruction allowed detailed analysis and measurement of both primary particle size and interconnecting pore sizes.

X-Ray diffraction analyses were undertaken on dry AMCP powders. The diffraction data were collected with a Bruker D8 Power Diffractometer using Cu K α1 radiation, a Ge primary monochromator and a Lynx Eye Detector. The scanning range was 5-90 degrees 2 theta at a speed of 0.5sec/step and a step size of 0.001 deg. Bruker Eva software was used to process the data with calibration peaks fitted to references in the ICDD (international centre for diffraction data) database.

**Trapping properties of synthetic AMCP**

Following synthesis, synthetic AMCP containing complex polysaccharides and/or protein (as described in section 1) were centrifuged at 1,500 rpm (5 mins) and supernatants discarded. After further two washes in pH 10 water particles were dissolved in 100 mM citric acid buffer (85.5 mM citric acid (Sigma-Aldrich), 15.5 mM sodium citrate tribasic dihydrate (Sigma-Aldrich), adjusted to pH 3) to release the organic material. Total protein content was measured using the Bradford protein assay (as per manufacturer’s protocol, Bio-Rad Laboratories, UK) while polysaccharide matter was quantified using a modified Periodic Acid Schiff (PAS; Jugdaohsingh R et al, 1999). This assay relies on the formation of a purple complex whose absorbance is proportional to the amount of polysaccharides in solution. Samples and standards (100 μl) were first incubated with 92 mM sodium periodate in 0.5 M sulphuric acid (10 μl/well, 37°C for 30 min with occasional shaking) and then with 2.7 % sodium arsenite in 0.68 M HCl (20 μl/well) at room temperature on a plate shaker (40 mins). Following the addition of Schiff’s reagent (50 μl/well; Merck KGaA, Germany) and a further 30 mins incubation at 37°C, the plate was read at 540 nm. Concentration of polysaccharides were determined against a standard curve containing the organic molecule of interest (0-250 μg/ml) and prepared in the lysate of AMCP particles to account for sample matrix.

Synthetic AMCP particles prepared in the presence of iron oxide nanoparticles (i.e. AMCP/Fe) were encapsulated in gelatine, embedded in the epoxy resin ‘Quetol 651’and sections cut to 70 nm thicknesses. Tomography experiments based on high-angle annular dark-field (HAADF) imaging in the STEM mode were performed at 200kV on a FEI Tecnai F20 electron microscope tilting the sample from -42° to +70° at 2° intervals about a single axis using a Fischione 2020 ultra-high-tilt tomography holder. Tilt series were aligned and reconstructed using Inspect3D software and AMIRA software was used for visualization.

**Uptake and internalisation of synthetic AMCP particles**

For the purpose of these experiments, leukocyte cones (n=4) were purchased from the National Blood Service, Cambridge, UK, and peripheral blood mononuclear cells (PBMC) isolated by density centrifugation with Lymphoprep (Axis Shield Diagnostics Ltd, Dundee, Scotland) as a separating medium. Briefly, blood was diluted with HBSS (Sigma-Aldrich) and 25 ml of diluted blood carefully layered over 10 ml Lymphoprep. After 20 min centrifugation (800 x g, brake off) the resulting layer of mononuclear cells was collected, washed three times in HBSS, and then re-suspended in R10 at 1.10^6^ cells/ml.

For flow cytometric analysis of particle uptake, 100 μl synthetic calcein labelled-AMCP were added to 1.10^6^ cells and incubated for 3 hrs before washing and re-suspending cells in ~200 μl ice cold PBS 1% BSA. Cells were then stained for 20 min on ice in the dark with PerCPCy5 conjugated CD14 (BD Biosciences). Cells were washed with ice cold PBS 1% BSA and re-suspended in a small volume of PBS containing 1% PFA and placed on ice in the dark until acquisition. Single stain compensation tubes as well as an unstained tube, were also prepared from cell samples to compensate for spectral overlap. Cells were acquired on a Cyan-ADP flow cytometer using Summit software for acquisition and analysis (Beckman Coulter), acquiring a minimum of 250,000 events per sample. An example of the analysis and gating strategy is shown in Figure S4. In brief, monocyte and lymphocyte gates were drawn based on forward and side scatter profiles, followed by CD14 gates (Figure. S4a), finally plotting calcein against forward scatter for analysis of calcein positivity of the lymphocyte and monocyte populations (Figure S4b).

For Imagestream analysis of particle internalisation and co-localisation with the lysosomal marker CD107a, 100 μl synthetic calcein-labelled AMCP were added to 1.10^6^ cells and incubated for 3 hrs before washing and re-suspending cells in ice cold PBS at 10^7^ cells in 100 μl. Cells were then stained for 20 minutes on ice in the dark with PerCPCy5 conjugated CD14 (BD Biosciences). Cells were washed and permeabilized before staining for 40 min with PE conjugated CD107a (BD Biosciences). After a final wash, cells were re-suspended in a small volume of PBS and filtered before acquisition on an ImagestreamX using INSPIRE software (Amnis), acquiring 25,000 events per sample. Appropriate single stained particle controls were synthesized, incubated with cells and acquired in addition to unstained particle/cell negative control and single surface marker controls required for the generation of a compensation matrix and analysis using IDEAS software (Amnis). An example of the analysis and gating strategy is shown in Figure S4. In brief, a single cell monocyte gate was drawn and used to gate highly focused cells. The single cell monocyte/Hi focus gate was then used to gate on the CD14^+^ cell population (Figure S5a). A further Calcein Hi gate was then drawn to identify the number of particle positive cells and used for further sub-analysis (Figure S5b). The internalization of particles for cells within the Calcein Hi gate was measured using internalization feature, using the CD14 stain to define the cell boundary (Figure S5c). Co-localization of CD107 and Calcein was analyzed for cells residing within the Calcium Hi gate, using the bright detail similarity feature. This feature is designed to quantify the co-localization of two probes within a defined region, increasing bright detail similarity scores indicate increases in the similarity of spatial location of the two probes (Figure S5d).

To further examine protein carriage by AMCP into cells, BSA, fluorescently tagged with Alexa Fluor 555 was used to track the incidence of protein within cells of the monocyte lineage in conjunction with fluorescently labelled AMCP. PBMC (n=3) were incubated with unlabeled AMCP, Calcein labelled AMCP, AMCP containing Alexa fluor 555 BSA (0.5mg/mL) and regular BSA (0.5mg/mL) or Calcein-labelled AMCP containing Alexa fluor 555 BSA (0.5mg/mL) and regular BSA (0.5mg/mL). After 3 hrs incubation with the various nanoparticle combination, cells were washed, stained for CD14, fixed (2 % PFA) and run on ImagestreamX (30,000 events acquired/sample). Double positive cells were assessed for the spatial similarity of the 2 fluorescent markers. The similarity feature used is a log transformed Pearson’s Correlation Coefficient and is a measure of the degree to which two images (one for each fluorophore) are linearly correlated within a specified region. Negative values indicate dissimilarity and the extent of similarity is indicated by increases in positive value.

**Visualisation of cellular AMCP nanoparticle digestion by live cell imaging of THP-1 macrophages**

To assess cellular digestion of synthetic nanoparticles by THP-1 macrophages, intracellular fluorescence was determined over time after incubation of the cells with calcein-labelled, fluorescent AMCP nanoparticles. Experiments were performed in technical triplicates. Briefly, the calcein stock solution (10 mg/ml in DMSO (Sigma-Aldrich)) was diluted 1:250 with phosphate solution prior to particle precipitation, for a final calcein concentration of 2 µg/ml after dilution of labelled nanoparticles to cell culture conditions. Negative controls included vehicle control (D10) and soluble calcein in D10 at 2 µg/ml. Following stimulation (3 hr, 37 °C/5 % CO_2_) THP-1 macrophages were washed three times in warm HBSS and were then replenished with phenol red-free D10 (phenol red-free DMEM (Sigma-Aldrich) supplemented with 10 % FCS, 100 U/ml penicillin, 100 μg/ml streptomycin, and 2 mM L-glutamine). Cells were visualised with the live content imaging device IncuCyteZOOM 2013A (Essen BioScience Ltd, UK), using phase-contrast and fluorescence channels (excitation 450-490 nm, emission 500-530 nm). Loss of calcein fluorescence was monitored for the following 24 hours by acquiring scans every two hours. Confluence percentage and count of discrete fluorescent objects were analysed using IncuCyteZOOM 2013A operating software. To address the potential issue of calcein quenching, a control acellular experiment was set up where soluble calcein was added to phenol red-free D10 at 10 or 0.1 µg/ml. As a positive control 1 mM iron hydroxide adipate tartrate (IHAT)^2^ was added to calcein solutions to induce iron-mediated calcein quenching. Mean fluorescence intensity of calcein solutions (± IHAT) was recorded under conditions equivalent to THP-1 uptake studies (i.e., with regards to well volume, culture plate and monitoring period).

**Biological properties of synthetic AMCP**

Inflammasome activation and cell viability

Studies looking at the inflammasome activation were carried out in PBMC isolated from 4 healthy volunteers, and as described in section 4. Following thawing and re-suspension in TCM, 1.10^6^ cells were first exposed to 10 ng/ml LPS (Sigma) and incubated for 3 hrs at 37 °C/95% air/5 % CO2. Cells were then washed and challenged with synthetic AMCP, vehicle (i.e. TCM) or with a positive control, namely crude peptidoglycan (*S.aureus*, Sigma) for another 3 hrs. After washing for 5 mins at 1,500 rpm, cells were then re-suspended in fresh TCM and left to incubate for another 21 hours. Supernatants were then collected and, as a surrogate marker for inflammasome activation^3^, were analysed for IL-β by ELISA according to manufacturer’s protocol (R&D system).

Experiments looking at the impact of synthetic AMCP on cell viability were assessed both in enriched monocytes and PMA-differentiated THP1. For monocytes studies, cells were incubated for 3 hrs with synthetic AMCP after which they were washed and re-suspended in fresh TCM. Following a chase of 21 hrs, cells were washed in DPBS (Sigma-Aldrich) prior to co-staining for monocyte marker CD14 (Alexa Fluor 488 conjugate, Becton Dickinson) and for cellular amines as surrogate marker for cellular permeability (Live/dead® Fixable Dead Cell Stain Kit, Violet dye, Invitrogen, Life Technologies Ltd, UK) using the recommended test volumes. Staining was performed on ice in the dark for 40 min (CD14) or 20 min (live/dead stain). Unstained and single stain compensation controls were set up alongside. Labelled cells were washed in PBS (Sigma-Aldrich) containing 1 % BSA (Heat shock 96 % standard powder; Fisher Scientific Ltd, UK) and were finally fixed in 250 µl DPBS containing 1 % paraformaldehyde. Data were acquired immediately on a Cyan ADP flow cytometer (Beckman Coulter, UK) with Summit software v4.3. Viability was determined as percentage of live (Violet^dim^) cells within a CD14^+^ monocyte gate and relative to control (i.e. cell treated with vehicle only). For differentiated THP1 studies, cells were challenged with synthetic AMCP or apatitic calcium phosphate ^3^ in duplicates and incubated for 24 hrs. Cell viability was then assessed by the lactate dehydrogenase assay, according to manufacturer’s protocol, and calculated as follows:

% cell viability = 100 – (100*((calcium phosphate OD- negative Control OD)/(cell death positive control- negative Control OD)))

Transcriptomics studies

PBMC isolated from 7 donors (leukocyte cones), as described in section 4, were subjected to a second density centrifugation to enrich for monocytes ^4^. In brief, the density medium Percoll (Sigma-Aldrich) was osmolarised to 285 mOsm by mixing 9.25 parts of Percoll with 0.75 parts of 10x DPBS (with calcium and magnesium; Sigma-Aldrich), and was then diluted to 46 % (v/v) in R10. The PBMC suspension was carefully layered over an equal volume of 46 % solution of 285 mOsm Percoll. After 30 min centrifugation (400 x g, brake off) the resulting layer of PBMC enriched in monocytes was collected, three times washed in HBSS and finally re-suspended at 1.10^6^ cells/ml in R10.

Culture and stimulation of monocyte-enriched PBMC were performed in sterile 15 ml Falcon tubes (Starlab UK Ltd, Milton Keynes, UK) at 1.10^6^ cells/ml and at 37 °C/ 5 % CO2. Freshly isolated monocyte-enriched PBMC were rested overnight, following which, they were replenished with fresh TCM (D10). 5.10^6^ cells were then stimulated for 3 hrs with vehicle D10 as negative control, synthetic AMCP nanoparticles or with a final concentration of 4 µg/ml soluble peptidoglycan (Source BioScience) either alone (sPg) or trapped within synthetic AMCP (AMCP/sPg). After challenge, stimulants were removed and cells washed twice in HBSS before being lysed with Nucleic Acid Purification Solution (Life Technologies Ltd, Paisley, UK) and stored at -80 °C prior to RNA isolation.

Total RNA was purified using an Abi Prism 6100 Nucleic Acid PrepStation (Applied Biosystems, UK) following manufacturer’s instructions and including a wash step with AbsoluteRNA wash solution (Applied Biosystems), again as per manufacturer’s instructions. Concentration and purity of eluted RNA were determined on a NanoDrop ND-1000 Spectrophotometer (Labtech International Ltd, UK). On occasions and to increase nucleotide concentration, a second purification procedure was performed using RNeasy MinEluteTM Cleanup Kit (Qiagen Ltd, Manchester, UK) according to manufacturer’s protocol. Samples were stored at -80 °C until further microarray processing.

Total RNA (100 ng) was labelled using an Ambion WT expression kit (Life Technologies, Bleiswijk, The Netherlands) and hybridized to human whole genome Genechip Human Gene 1.1 ST arrays coding 19,732 genes, (Affymetrix, Santa Clara, CA). Sample labelling, hybridization to chips and image scanning was performed according to manufacturer's instructions.

Microarray analysis was performed using MADMAX pipeline for statistical analysis of microarray data ^5^. Quality control was performed and all arrays met our criteria. For further analysis a custom annotation was used based on reorganized oligonucleotide probes, which combine all individual probes for a gene ^6^. Only genes that had at least five probes present on the array were taken into account. Expression values were calculated using robust multichip average (RMA) method, which includes quantile normalisation^7^. Microarray data were filtered, and probe sets with expression values higher than 20 on more than four arrays were considered to be expressed and selected for further statistical analysis. In addition, an Inter Quartile Range (IQR) cut-off of 0.25 was used to filter out genes that showed little variation between the conditions. Significant differences in expression were assessed using paired Intensity-Based Moderated T-statistic (IBMT) ^8^.

To assess similarity of gene regulation by peptidoglycan in soluble (sPg) and particulate form (AMCP/sPg) only genes that were significantly changed (p < 0.01) by at least one of the two treatments were considered. For those, the average signal log ratios (SLR; log_2_ fold change) of each stimulant compared to control treatment were calculated and visualised in a correlation plot. All microarray data are MIAME compliant and have been submitted to the Gene Expression Omnibus (accession number [GSE59498](http://www.ncbi.nlm.nih.gov/geo/query/acc.cgi?token=gtmvoisivhaznmh&acc=GSE59498)).

Surface PD-L1 expression

Studies looking at PD-L1 expression were carried out in PBMC isolated from 7 healthy volunteers. Following thawing and re-suspension in TCM, cells were challenged for 3 hrs with either Gram^+^ peptidoglycan at 5 µg/ml (*S.aureus*, Sigma), synthetic calcein labelled-AMCP containing Gram^+^ peptidoglycan equivalent to 5ug/mL, synthetic calcein labelled-AMCP alone or left unstimulated (negative control). After washing for 5 mins at 1,500 rpm, cells were then re-suspended in fresh TCM and left to incubate for another 21 hrs. Following stimulation, cells were washed with ice cold phosphate buffered saline (PBS) containing 1% BSA and stained with PD-L1 PE (Biolegend, US) and CD11b PE-Cy7 (Biolegend, US) according to the manufacturers’ instructions for 30 min on ice in the dark. Following staining, cells were washed re-suspended in a small volume of PBS containing 1% paraformaldehyde and placed on ice in the dark until analysis. Single stain compensation tubes for each stain used, as well as an unstained tube, were also prepared to compensate for spectral overlap. Cells were filtered immediately prior to acquisition on a Cyan-ADP flow cytometer that used Summit software (both from Beckman Coulter, UK) for acquisition and data analysis. A minimum of 500,000 events per sample were acquired. Remaining sample (n = 3 subjects) were analysed on an ImagestreamX using INSPIRE software (both from Amnis, USA), acquiring a minimum of 10,000 events per sample. Compensation matrices were generated by running single stained particles or cells (i.e., single cell surface marker) and analysed using IDEAS software (Amnis, USA).

**Supplementary data**

**Supplementary Table 1:** **Descriptive statistics of the synthetic AMCP particle size distributions measured by nanoparticle tracking analysis.**

|  |  | D10  (nm; n=3 ±SD) | | D50  (nm; n= 3±SD) | D90  (nm; n=3-5 ±SD) | Average  (nm; n= 3±SD) |
| --- | --- | --- | --- | --- | --- | --- |
| **Cell culture conditions t=0** | **AMCP** | 54 ± 7 | | 98 ± 62 | 213 ± 59 | 118 ± 24 |
|  | **AMCP/sPg** | 52 ± 4 | | 93 ± 7 | 199 ± 6 | 113 ± 7 |
|  |  |  | |  |  |  |
| **Cell culture conditions t=3** | **AMCP** | 53 ± 4 | | 99 ± 14 | 215 ± 42 | 119 ± 18 |
|  | **AMCP/sPg** | 51 ± 2 | | 92 ± 4 | 184 ± 7 | 107 ± 4 |
|  |  |  | |  |  |  |
|  |  | |  |  |  |  |

AMCP: Amorphous calcium phosphate with a model protein (bovine serum albumin) incorporated.

AMCP/sPg: AMCP with the further incorporation of peptidoglycan

D10, D50 and D90 are sizes calculated with linear interpolation using the two data points above and below the 10^th^, 50^th^ and 90^th^ percentile, respectively.

See Methods for detailed synthesis.

**Supplementary Figure 1:** X-ray diffraction patterns of calcium phosphate nanoparticles prepared in (a) the absence of both magnesium (Mg) and bovine serum albumin (BSA), (b) in the presence of 0.9mM (Mg; final concentration), (c) 1.8 mm Mg, (d) 1.8 mM Mg and BSA, (e) 3.6 mM Mg and (f) 3.6 mM Mg and BSA. Reference peaks in red correspond to hydroxyapatite.

**
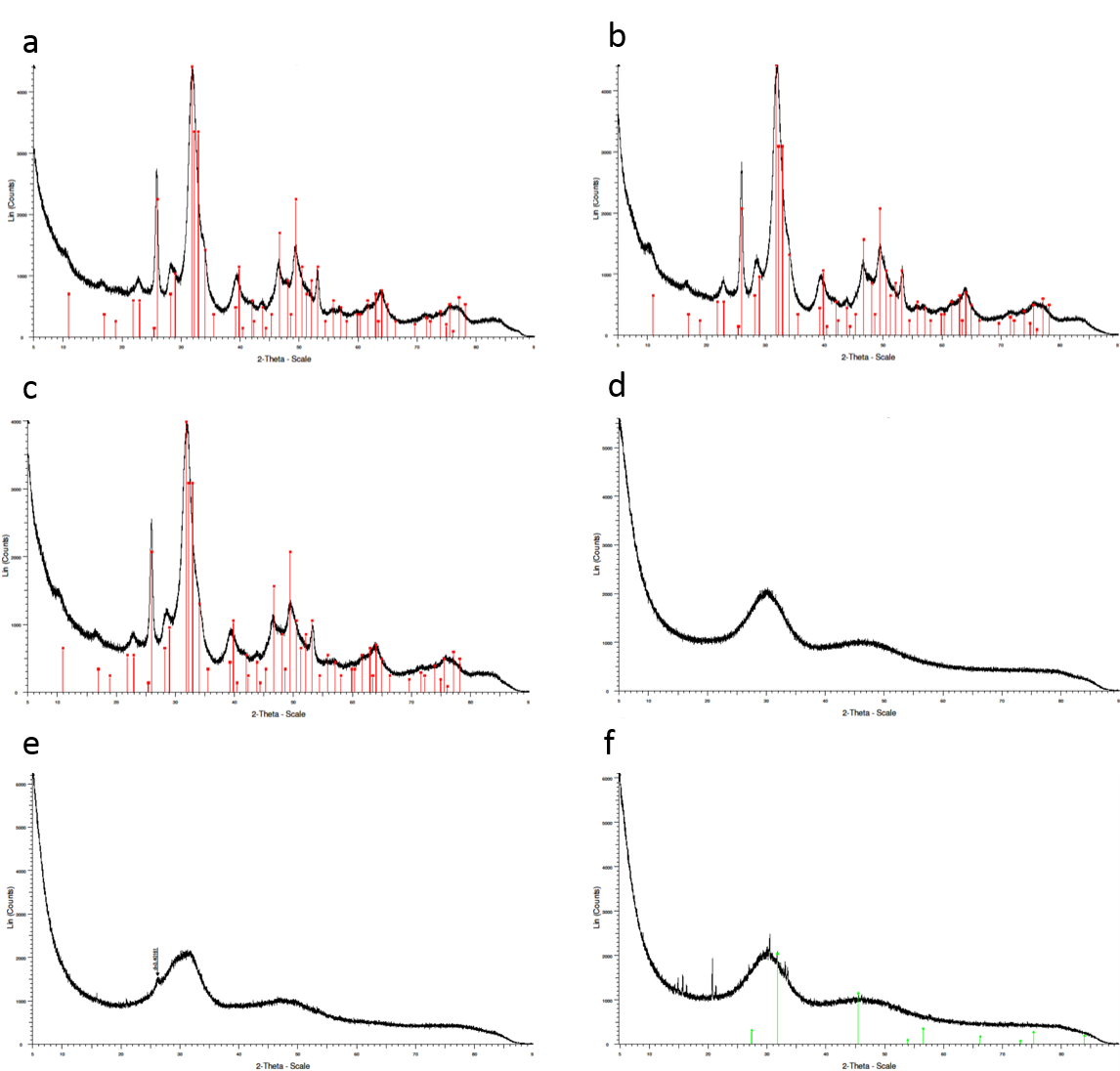
**

**Supplementary Figure 2: A.** Percentage incorporation, from the original solution of fluorescent protein (Texas Red OVA and BSA 555) in synthetic AMCP. Comparisons of percentage incorporation of **B**- OVA or **C**- BSA 555 in synthetic AMCP (red) or regular ACP (black). n= 3 experiments ± SD.

**Supplementary Figure 3:** *PD-L1* gene (*CD274*) up-regulation by sPg and AMCP/sPg, expressed as log2 fold difference compared to unstimulated control (n=7).

**Supplementary Figure 4: Flow Cytometry gating strategy and analysis. a.** Example dotplot showing monocyte (R1) and Lymphocyte (R14) gates drawn based on FSC and SSC profile of PBMC, followed by histogram plots of CD14+ fluorescence intensity with gates for positive (R2) and negative populations (R15) of respective (monocyte and lymphocyte) cell populations and final FSC versus Calcein dotplots used for analysis of sequentially gated populations. **b**. Example dotplots of analysis comparing Calcein positivity of gated CD14+ Monocytes and CD14- Lymphocytes incubated with and without AMCP.


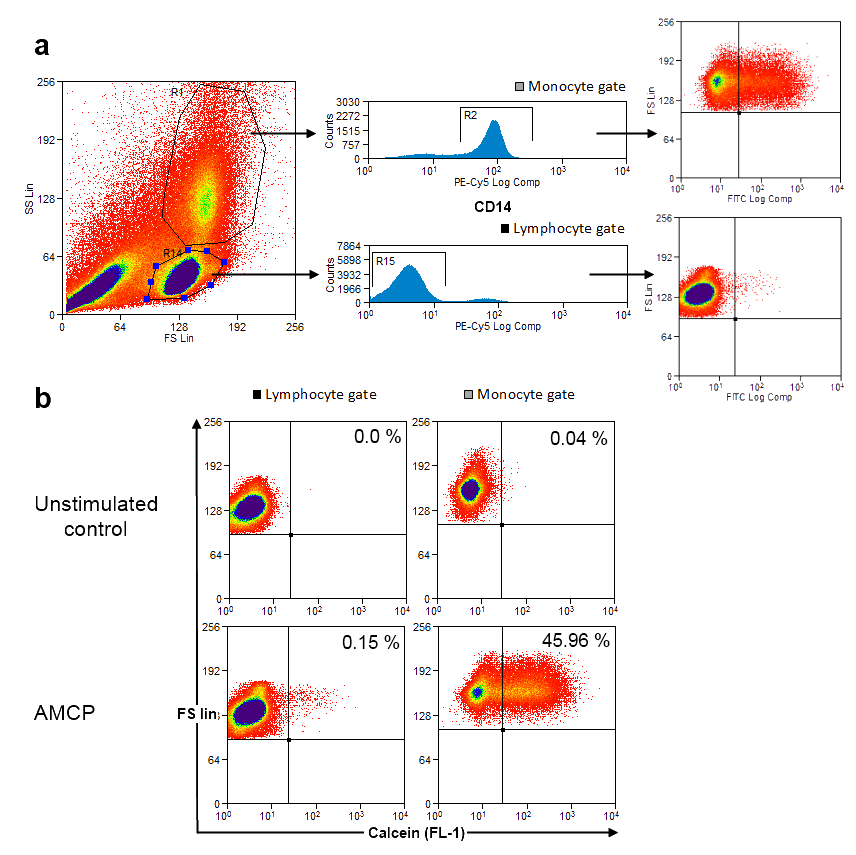


**Supplementary Figure 5: Imagestream gating strategy and analysis. a.** Example Plots showing the single cell monocyte gate, followed by the focused and CD14+ cell sequential gating. **b**. Example dotplots of Calcein stain intensity versus max pixel used to obtain Calcein Hi gated cells, plots show a negative control versus AMCP. **c**. Example histograms of Internalization score sub-analysis of Calcein Hi Gated cells over time obtained using the CD14+ stain to determine the cell boundary **d**. Example histograms of the bright detail similarity score sub-analysis of Calcein Hi gated cells for CD107 and Calcein over time. **e**. Example images of cells residing within the Hi Similarity gate exhibiting internalized nanoparticles and co-localization of CD107 and Calcein stains.

**
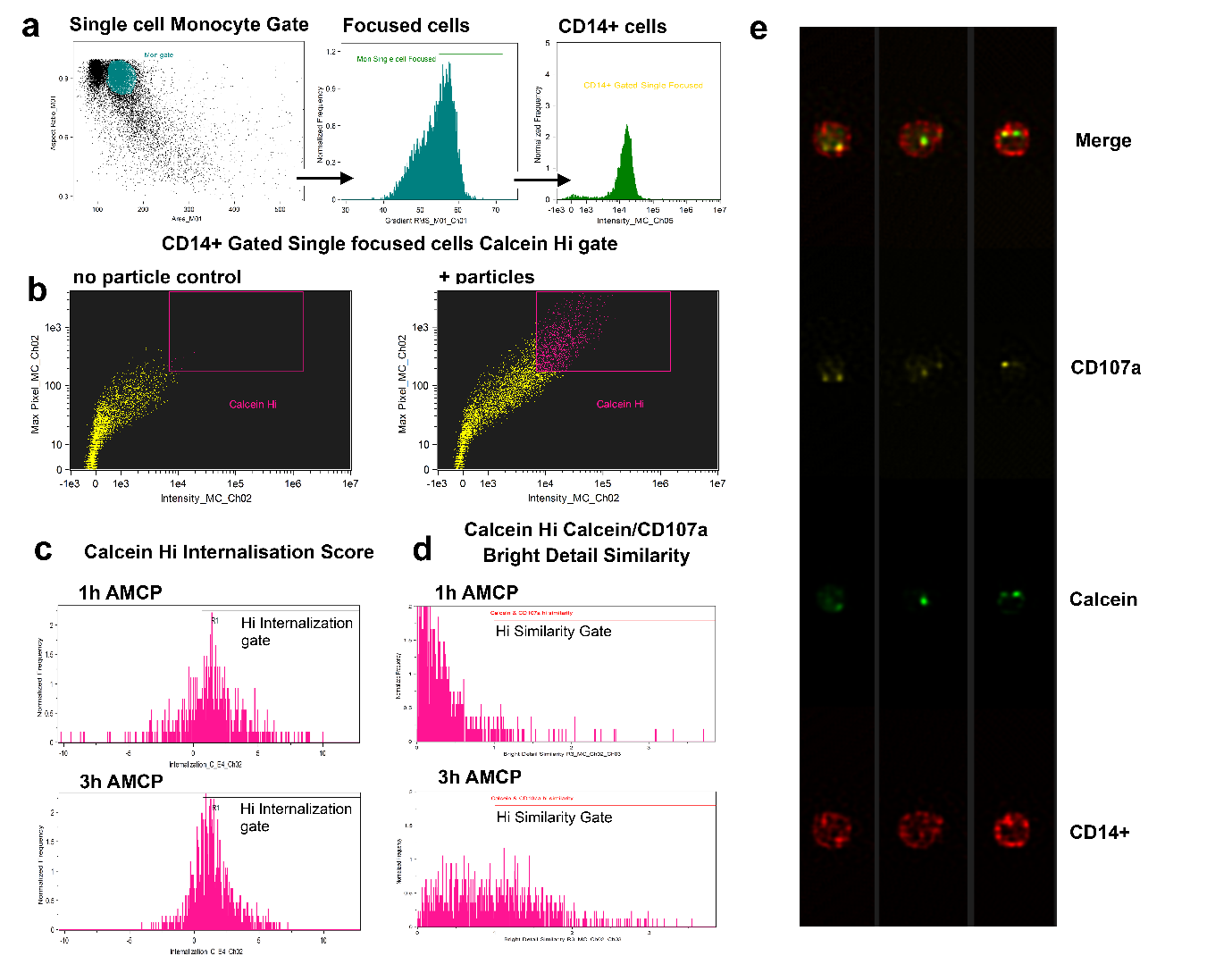
**

1. Boskey, A. L.; Posner, A. S., Magnesium Stabilization of Amorphous Calcium-Phosphate - Kinetic Study. *Materials Research Bulletin* 1974**,** **9** (7), 907-916.

2. Powell, J. J.; Bruggraber, S. F.; Faria, N.; Poots, L. K.; Hondow, N.; Pennycook, T. J.; Latunde-Dada, G. O.; Simpson, R. J.; Brown, A. P.; Pereira, D. I., A nano-disperse ferritin-core mimetic that efficiently corrects anemia without luminal iron redox activity. *Nanomedicine* 2014.

3. Pele, L.; Haas, C. T.; Hewitt, R.; Faria, N.; Brown, A.; Powell, J., Artefactual nanoparticle activation of the inflammasome platform: in vitro evidence with a nano-formed calcium phosphate. *Nanomedicine (Lond)* 2014, 1-12.

4. Martinez, F. O., Analysis of gene expression and gene silencing in human macrophages. *Curr Protoc Immunol* 2012**,** **Chapter 14**, Unit 14 28 11-23.

5. Lin, K.; Kools, H.; de Groot, P. J.; Gavai, A. K.; Basnet, R. K.; Cheng, F.; Wu, J.; Wang, X.; Lommen, A.; Hooiveld, G. J.*, et al.*, MADMAX - Management and analysis database for multiple ~omics experiments. *J Integr Bioinform* 2011**,** **8** (2), 160.

6. Dai, M.; Wang, P.; Boyd, A. D.; Kostov, G.; Athey, B.; Jones, E. G.; Bunney, W. E.; Myers, R. M.; Speed, T. P.; Akil, H.*, et al.*, Evolving gene/transcript definitions significantly alter the interpretation of GeneChip data. *Nucleic Acids Res* 2005**,** **33** (20), e175.

7. Bolstad, B. M.; Irizarry, R. A.; Astrand, M.; Speed, T. P., A comparison of normalization methods for high density oligonucleotide array data based on variance and bias. *Bioinformatics* 2003**,** **19** (2), 185-193.

8. Sartor, M. A.; Tomlinson, C. R.; Wesselkamper, S. C.; Sivaganesan, S.; Leikauf, G. D.; Medvedovic, M., Intensity-based hierarchical Bayes method improves testing for differentially expressed genes in microarray experiments. *BMC Bioinformatics* 2006**,** **7**, 538.
